# Uterine fibroids and longitudinal profiles of the vaginal microbiota in a cohort presenting for transvaginal ultrasound

**DOI:** 10.1371/journal.pone.0296346

**Published:** 2024-02-05

**Authors:** Sarah J. Robbins, Sarah E. Brown, Christina A. Stennett, Susan Tuddenham, Elizabeth D. Johnston, Amelia M. Wnorowski, Jacques Ravel, Xin He, Katrina S. Mark, Rebecca M. Brotman

**Affiliations:** 1 Department of Epidemiology and Public Health, University of Maryland School of Medicine, Baltimore, Maryland, United States of America; 2 Institute for Genome Sciences, University of Maryland School of Medicine, Baltimore, MD, United States of America; 3 Department of Medicine, Division of Infectious Diseases, Johns Hopkins University School of Medicine, Baltimore, Maryland, United States of America; 4 Department of Obstetrics and Gynecology, University of Maryland School of Medicine, Baltimore, Maryland, United States of America; 5 Diagnostic Radiology and Nuclear Medicine, University of Maryland School of Medicine, Baltimore, Maryland, United States of America; 6 Department of Epidemiology and Biostatistics, University of Maryland, College Park, Maryland, United States of America; GGD Amsterdam, NETHERLANDS

## Abstract

Bacterial vaginosis, characterized in part by low levels of vaginal *Lactobacillus* species, has been associated with pro-inflammatory cytokines which could fuel uterine fibroid development. However, prior work on the associations between uterine fibroids and vaginal bacteria is sparse. Most studies have focused on assessment of individual taxa in a single sample. To address research gaps, we sought to compare short, longitudinal profiles of the vaginal microbiota in uterine fibroid cases versus controls with assessment for hormonal contraceptives (HCs), a possible confounder associated with both protection from fibroid development and increases in *Lactobacillus*-dominated vaginal microbiota. This is a secondary analysis of 83 reproductive-age cisgender women who presented for transvaginal ultrasound (TVUS) and self-collected mid-vaginal swabs daily for 1–2 weeks before TVUS (Range: 5–16 days, n = 697 samples). Sonography reports detailed uterine fibroid characteristics (N = 21 cases). Vaginal microbiota was assessed by 16S rRNA gene amplicon sequencing and longitudinal microbiota profiles were categorized by hierarchical clustering. We compared longitudinal profiles of the vaginal microbiota among fibroid cases and controls with exact logistic regression. Common indications for TVUS included pelvic mass (34%) and pelvic pain (39%). Fibroid cases tended to be older and report Black race. Cases less often reported HCs versus controls (32% vs. 58%). A larger proportion of cases had low-*Lactobacillus* longitudinal profiles (48%) than controls (34%). In unadjusted analysis, *L*. *iners*-dominated and low-*Lactobacillus* profiles had higher odds of fibroid case status compared to other *Lactobacillus*-dominated profiles, however these results were not statistically significant. No association between vaginal microbiota and fibroids was observed after adjusting for race, HC and menstruation. Results were consistent when number of fibroids were considered. There was not a statistically significant association between longitudinal profiles of vaginal microbiota and uterine fibroids after adjustment for common confounders; however, the study was limited by small sample size.

## Introduction

Uterine leiomyomas (fibroids) are benign, smooth-muscle tumors of the uterus [1] and are hormonally regulated by estrogen and progesterone [2]. The incidence of fibroids increases after menarche and resolves after menopause [3]. It is estimated that more than 80% of Black women and 70% of white women develop fibroids by 50 years of age, with Black women having an earlier age of onset [4]. Fibroids are the most frequent indication for hysterectomy in the U.S., generating direct medical costs of up to $9 billion annually [5]. Among those with fibroids, 30% experience severe symptoms such as abnormal uterine bleeding, pelvic pain, anemia, digestive and bladder issues, and infertility or obstetrical complications [6, 7]. In addition, fibroids are associated with an increased risk of birth complications such as miscarriage, preterm delivery, cesarean section, and postpartum hemorrhage [8]. Those with fibroids often report significant distress, including anxiety and depression [9–11]. Although fibroids are common and have high associated healthcare costs, their etiology remains largely unknown.

Epidemiologic studies have identified a number of factors associated with a greater risk for fibroids, including older age, African American race, early age of menarche, nulliparity, and adolescent talc use (with or without douching) [12–18]. One theorized etiology of fibroids is that injury resulting from reproductive tract infections, such as *Chlamydia trachomatis* (CT), may contribute to fibroid risk as inflammation can promote increased smooth muscle cell proliferation and production of extracellular matrix, factors in tumor cell progression. However, data supporting this hypothesis are sparse and inconsistent [19–21]. It is plausible that bacterial vaginosis (BV), and the associated non-optimal (low-*Lactobacillus*) vaginal microbiota, may increase risk for fibroid development with mechanisms also related to inflammation in the reproductive tract. BV is characterized by a vaginal microbiota with a low abundance of vaginal *Lactobacillus* spp. and a higher abundance of strict and facultative anaerobic bacteria such as *Gardnerella*, *Atopobium*, *Prevotella* and *Streptococcus* spp [22]., and it has been associated with increases in levels of pro-inflammatory cytokines and chemokines measured in cervicovaginal secretions [22–26]. In an optimal vaginal microbiota, *Lactobacillus spp*. produce lactic acid [32, 33], a key metabolite that provides protection against pathogens through a number of mechanisms including, acidifying the vagina, modulating host epithelial functions and regulating host immune response [25, 26]. Other factors may be linked to both the vaginal microenvironment and uterine fibroids. For instance, hormonal contraception (HC) has been associated with the suppression of both innate and adaptive components of immunity in the vagina [27], decreased BV [28], and reduced risk for uterine fibroids [29–31].

Three epidemiologic studies from Moore *et al*. provided formative data on the associations between BV, vaginal microbiota and fibroid development. In a prospective cohort study of reproductive-age African American women in Detroit, Moore *et al*. found that self-reported history of BV was associated with fibroid incidence; [32] however, in follow-up studies, the investigators reported neither BV-associated bacterial taxa [33] nor Nugent-BV (BV assessed by microscopy with Nugent’s Gram stain score [34]) were associated with incident uterine fibroids [35]. These studies focused on a single vaginal sample for BV exposure assessment, and fibroid incidence was measured over a 5-year follow-up period.

We sought to expand upon prior findings by comparing longitudinal profiles of the vaginal microbiota in those with and without uterine fibroids. Short longitudinal profiles of the vaginal microbiota provide a comprehensive spectrum as the vaginal microbiota can fluctuate rapidly for some individuals [36–39]. We also sought to include HC use in the analysis as it may influence both fibroid development and vaginal microbiota composition.

## Methods

### Study design and population

This study was a secondary analysis of 83 reproductive-age cisgender women recruited to the Gynecology and Lubricant Effects (GALE) study, a longitudinal general health study in Baltimore, MD, which assessed the effect of lubricant used during transvaginal ultrasound (TVUS) on the vaginal microbiome [40]. Non-pregnant patients scheduling a transvaginal ultrasound (TVUS) at the Diagnostic Radiology and Nuclear Medicine Department at the University of Maryland Medical Center between May 2017 and March 2020 were contacted in advance of their appointment and offered recruitment to the parent study [40].

Participants were referred to TVUS for a number of conditions, including pelvic mass (i.e. fibroids, cysts, adenomyosis), localization of intrauterine device, abnormal uterine bleeding, screening for malignancy, and pelvic pain. Sonography reports from TVUS collected information on the number, type, location and size of fibroids, as well as information on additional findings including adenomyosis, cysts, PID, cancer and other conditions [41]. Participants were excluded from the parent study if they were under age 18, diagnosed with pelvic inflammatory disease, cancers of the uterus, ovaries, or pelvic structures, prescribed antibiotics or antifungals within a month of starting the study, had an immune condition, were taking immunosuppressants, or self-reported lubricant use in the week prior to TVUS. At baseline, participants underwent a cervical exam and blood draw. Participants were also excluded if laboratory results confirmed *Neisseria gonorrhea*, CT or *Trichomonas vaginalis* (BD MAX CT/GC/TV), HIV (Abbot ARCHITECT HIV Ag/Ab Combo), syphilis (BD Macro-Vue), pregnancy or were diagnosed with symptomatic BV or vulvovaginal candidiasis (VVC) at the baseline visit of the parent study. Those diagnosed with symptomatic BV at the baseline clinic visit were prescribed antibiotics and were therefore excluded from the parent study. Individuals were then included in this secondary analysis if they met the inclusion and exclusion criteria of the parent study and were premenopausal (defined by the STRAW criteria [42]). This provided a case-control of 21 cases with fibroids detected on TVUS and 62 controls with no fibroids detected. Controls were included even if they had other findings (cysts and adenomyosis). A sensitivity analysis of controls with no clinical findings (n = 32) was also conducted.

This secondary analysis was based on mid-vaginal samples collected once daily for approximately 1–2 weeks prior to the patient’s TVUS appointment. Mid-vaginal samples were collected at the baseline visit by a clinician and the remaining samples were self-collected. Self-collected mid-vaginal swabs are not distinguishable in bacterial composition from clinician-collected samples [43]. Each participant had 5 to 16 samples available (average: 8 samples). Individuals were excluded if they collected less than 5 mid-vaginal samples before TVUS (N = 2). Taxa present at less than 10^−5^ across all samples were removed from this analysis, providing 224 taxa for the final dataset. In addition, samples with less than 500 reads (n = 5) were excluded. Sensitivity analyses dropping samples with less than 1,000 reads (additional n = 2) also did not affect results. Participants also completed demographic and health behavior surveys at enrollment, and brief online daily diaries indicating menstruation, vulvovaginal symptoms and personal behaviors every 24 hours. Data on HC use was collected at baseline, including the type and consistency of use. Covariates were obtained from surveys and daily diaries, which were administered with REDCap, a Web-based application for constructing surveys [44, 45]. This study was approved by the University of Maryland, Baltimore (UMB) Institutional Review Board, and informed written consent was obtained from each participant.

### Laboratory procedures

Participants self-collected mid-vaginal swabs (Copan ESwabs in 1ml of Amies Transport Medium only (N = 5 participants), 1 mL Amies and 1 mL RNALater (N = 1 participant), or 1 mL Amies and 1 mL modified C2 (N = 77 participants)) and stored the samples in their home freezer (-20°C). Samples were transported back to the research site in coolers and were subsequently archived at -80°C. DNA was extracted from Vaginal Eswabs with the MagAttract Microbial DNA kit (Qiagen, Hilden, GER) using a custom automated protocol on the Hamilton Microlab Star instrument. High-throughput sequencing of V3-V4 hypervariable regions of 16S rRNA genes was conducted using the Illumina HiSeq or MiSeq platform [46]. Both platforms have demonstrated complete within-woman agreement in the classification of vaginal bacterial communities (κ = 1.0) [46]. Sequence data processing was carried out using dada2 integrated into a custom-designed bioinformatics pipeline [47]. Taxonomy of amplicon sequence variants was assigned by the RDP Naïve Bayesian Classifier [48] trained with the SILVA v128 16S rRNA gene database [49], and major vaginal taxa were assigned species-level annotations using speciateIT [50].

### Exposure and outcomes

The primary exposure assessed in this secondary data analysis was each participant’s longitudinal profiles of their vaginal microbiota. The vaginal microbiota from a single mid-vaginal sample can be classified into broad categories called community state types (CSTs) [51]. There are five main CSTs that have been previously identified in the literature [51, 52]. Four CSTs are dominated by *Lactobacillus* species (CST I: *L*. *crispatus*, CST II: *L*. *jensenii*, CST III: *L*. *iners*, and CST V: *L*. *gasseri*), but CST IV (termed molecular-BV [22]) has a higher abundance of strict or facultative anaerobic bacteria, as well as a low abundance of *Lactobacillus* spp [36, 51, 53–64]. We first classified the microbiota from each mid-vaginal sample (697 samples prior to TVUS) into CSTs. CSTs were assigned using VALENCIA, a classification algorithm based on the similarity to the centroid of each CST determined from a large reference set of over 13,000 vaginal samples [65]. Hierarchal clustering of these CSTs was then used to assign longitudinal profiles to each participant. Due to the small sample size, the longitudinal profiles of vaginal microbiota were condensed to three categories [optimal *Lactobacillus* (consisting of (1) *L*. *crispatus*-, *L*. *jensenii*-, and *L*. *gasseri*-dominated), (2) *L*. *iners*-dominant and (3) low*-Lactobacillus* vaginal microbiota]. We considered *L*. *iners* as a separate category as prior work indicates a *L*. *iners*-dominated vaginal microbiota may be less optimal than those predominated by *L*. *crispatus*, *L*. *jensenii*, or *L*. *gasseri*, but *L*. *iners* dominance appears to remain more protective compared to low-*Lactobacillus* microbiota containing BV-associated bacteria [66, 67].

The primary outcomes assessed in this analysis were fibroid detection and the number of fibroids detected. Information on fibroid outcomes was obtained from sonography reports completed by a study radiologist. Fibroid detection was defined as a dichotomous variable (yes/no). Number of fibroids was categorized as no fibroids, 1 to 2 fibroids, or 3 or more fibroids based on how the question was structured on the sonography report. Age was assessed continuously. Contraception was categorized as hormonal, non-hormonal, and none for modeling, although sensitivity analyses were conducted to confirm whether a four-category HC variable (progestin-only, combined hormonal, non-hormonal and no contraception) or binary categorization (HC versus non-HC) affected the association of the vaginal microbiota with fibroids. Race was self-defined as either Asian, Native American or Alaskan Native, Native Hawaiian or Pacific Islander, Black or African American, white or other, and due to small sample size was dichotomized for modeling purposes to Black or African American and either white or Asian. One individual self-reported Native American or Alaskan Native race and they were combined with white. Menstruation was assessed in numerous ways, including a variable indicating whether the participant reported menstrual bleeding to the daily diaries on the same days as the mid-vaginal sampling and a participant’s typical severity of menstrual bleeding (options included heavy, moderate, light and not applicable) reported at baseline. An indication for referral to TVUS for abnormal uterine bleeding was also tested as a potential confounder.

### Statistical analysis

We conducted a case-control study comparing longitudinal profiles of the vaginal microbiota among all reproductive-age participants in the GALE study with uterine fibroid data available. Bivariate analyses were conducted to identify potential confounders using demographic and behavioral variables. Chi-square and Fisher’s exact test for categorical variables and Wilcoxon rank sum test for continuous variables were used to assess the association of covariates with condensed longitudinal profiles of the vaginal microbiota and the detection of fibroids. Statistical significance was defined as p<0.05. Important confounders, including type of contraception and menstrual bleeding during time of mid-vaginal sampling, were controlled for *a priori* given their known association with the vaginal microbiota and fibroids in the literature [28–31, 38, 41, 68–70, 71–76]. To assess the association between condensed longitudinal profiles of the vaginal microbiota and fibroid detection, exact logistic regression was used to estimate odds ratios (ORs) with 95% confidence intervals (CIs), and exact multinomial logistic regression was used to assess the association between condensed longitudinal profiles of the vaginal microbiota and fibroid number (none, 1–2 fibroids and 3 or more fibroids). In all modeling, the reference group was no fibroids. Confounders were assessed by adding covariates one at a time to the model to determine whether they altered the main point estimate by more than 10%. Models were adjusted for race (binary variable), type of contraception, and menstrual bleeding during sampling. All analyses were performed using SAS OnDemand for Academics (SAS Institute Inc., Cary, North Carolina).

## Results

### Population characteristics

Among the 83 reproductive-age participants included in this case-control study, the median age was 32 years (IQR: 27–37), 60% were Black, 49% reported HC use, 41% had an annual income of equal to or greater than $61,000 and 50% had less than or equivalent to high school education. Indication for TVUS among both cases and controls was most often for assessment of pelvic pain (52% vs 34%) or pelvic mass (43% vs 31%), and the most common TVUS finding among controls was no significant finding (52%). Among GALE study participants, 25% had fibroids detected during TVUS. Of those diagnosed with fibroids (n = 21), most had 1 to 2 fibroids (57%), fibroids measuring 2 to 4 centimeters in diameter (52%), multiple types of fibroids (48%) and either multiple locations of fibroids (43%) or fibroids located in the uterine corpus (43%).

**Fig 1:**
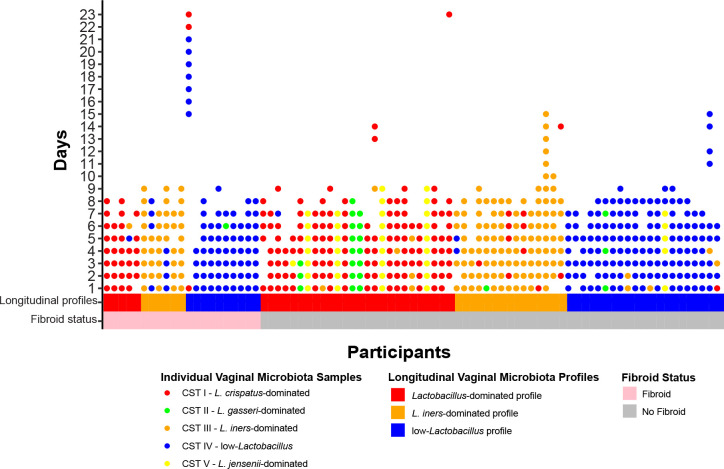
Condensed longitudinal profiles of the vaginal microbiota of 83 participants (21 cases and 62 controls). Each column represents a participant, and each dot represents a single mid-vaginal sample classified into a CST. Condensed longitudinal profiles are presented by fibroid status (horizontal bar). The CSTs (collected daily) of each participant are shown grouped into categories of condensed longitudinal profiles. Condensed longitudinal profiles are also stratified by fibroid status.

The distribution of the vaginal microbiota longitudinal profiles was similar between cases and controls (Table 1 and Fig 1). Clustering revealed 5 longitudinal profiles: *Lactobacillus jensenii*-dominated, *Lactobacillus gasseri*-dominated, *Lactobacillus iners*-dominated, *Lactobacillus crispatus*-dominated and low-*Lactobacillus* (S1 Fig). Characteristics significantly associated with fibroids included older age and Black race. The majority of controls reported HC use (58%) versus 32% of cases, although four individuals did not respond to the survey question about contraception use. At baseline, cases most commonly reported moderate bleeding during a typical menstrual cycle (47%), while most controls reported heavy bleeding (43%). Many participants were indicated for TVUS for abnormal uterine bleeding (19% and 26%, cases and controls respectively). Black race, low income and menstrual bleeding during sampling were all independently associated with a low-*Lactobacillus* longitudinal profile (all p<0.05).

**Table 1:** Population characteristics of 83 reproductive-age participants with and without uterine fibroids detected at TVUS in an observational study in Baltimore, MD, 2017–2020.

Variables	Fibroids	No Fibroids	p-value
	N = 21 (column %)	N = 62 (column %)	
**Longitudinal Characteristics**			
**Vaginal microbiota *longitudinal* profiles**			0.66^a^
*L*. *crispatus*-dominated	5 (24)	20 (32)	
*L*. *gasseri*-dominated	0 (0)	2 (3)	
*L*. *iners*-dominated	6 (28)	15 (24)	
Low-*Lactobacillus*	10 (48)	21 (34)	
*L*. *jensenii*-dominated	0 (0)	4 (7)	
**Menstrual bleeding during sampling**			0.66^b^
Yes	11 (52)	29 (47)	
No	10 (48)	33 (53)	
**Baseline Characteristics**			
**Age (years) median [IQR]**	37 [35–42]	31 [26–35]	**<0.01** ^**c**^
**Race** ^**1**^			**0.02** ^**a**^
Asian	1 (5)	4 (6)	
Native Hawaiian or Pacific Islander	0 (0)	0 (0)	
Native American or Alaskan native	0 (0)	1 (2)	
Black or African American	18 (86)	31 (51)	
White	2 (9)	25 (41)	
**Education** ^**1**^			0.44^b^
Less than or equivalent to high school	13 (62)	28 (46)	
Associate or bachelor’s degree	5 (24)	22 (36)	
Graduate or professional degree	3 (14)	11 (18)	
**Income** ^**4**^			0.85^b^
<$25K	5 (26)	17 (28)	
$25-59K	7 (37)	18 (30)	
≥$60K	7 (37)	25 (42)	
**Frequency of cigarette smoking in the prior 2 months** ^**2**^			>0.99^a^
Daily	2 (10)	5 (8)	
Weekly	0 (0)	0 (0)	
None	18 (90)	56 (92)	
**Frequency of alcohol use in the prior 2 months**^**3**^			0.75^a^
Several times a week or every day	4 (20)	13 (22)	
Several times a month or about once a `week	7 (35)	14 (23)	
Once a month or less	5 (25)	21 (35)	
Never	4 (20)	12 (20)	
**Severity of menstrual bleeding** ^**4**^			0.25^**a**^
Heavy	5 (33)	16 (43)	
Moderate	7 (47)	11 (30)	
Light	0 (0)	6 (16)	
Not applicable	3 (20)	4 (11)	
**Indicated to have TVUS for abnormal uterine bleeding**			0.53^b^
Yes	4 (19)	16 (26)	
No	17 (81)	46 (74)	
**Contraception** ^**4**^			0.12^a^
Progestin-only	4 (21)	27 (45)	
Combined	2 (10)	8 (13)	
Non-hormonal	6 (32)	16 (27)	
None	7 (37)	9 (15)	
**Pregnancy history** ^**4**^			0.74^a^
Full-term pregnancy	9 (56)	29 (56)	
Early pregnancy loss only	2 (13)	3 (6)	
Never been pregnant	5 (31)	20 (38)	

Abbreviations: IQR, interquartile range; TVUS, transvaginal ultrasound.

Statistical tests used included: (a) Fisher’s exact test, (b) Chi-square test, (c) Wilcoxon two-sample test

1. Missing for 1 participant

2. Missing for 2 participants

3. Missing for 3 participants

4. Missing more than 3 participants

### Modeling

In unadjusted models, *L*. *iners*-dominated (OR = 2.04; 95% CI: 0.44–10.11) and low-*Lactobacillus* (OR = 2.44; 95% CI: 0.64–10.59) longitudinal profiles had higher odds of presence of fibroids versus controls in comparison to *Lactobacillus-*dominated profiles, although the findings were not statistically significant (Table 2). Findings were null in the models adjusted for race, type of contraception, and menstrual bleeding during sampling. Results were also similar when we assessed number of fibroids and collapsed categories for small sample sizes (dichotomized *Lactobacillus-*dominated versus low-*Lactobacillus*, and restricted to HC versus non-HC). Results also remained consistent when controls were restricted to those with no clinical findings on TVUS (n = 32).

**Table 2:** Association between condensed longitudinal profiles of the vaginal microbiota with fibroid detection and number of fibroids among reproductive-age participants, Baltimore, MD, 2017–2020.

**Longitudinal Vaginal Microbiota Profiles**		**Fibroid Presence**			**OR (95% CI)**	
		**Fibroid**		**No Fibroids**		
		**N (column %)**		**N (column %)**		
**Unadjusted**^**a**^ **(N = 83)**						
**Vaginal microbiota**						
*Lactobacillus-*dominated		5 (24)		26 (42)	REF	
*L*. *iners*-dominated		6 (29)		15 (24)	2.05 (0.44, 10.11)	
Low-*Lactobacillus*		10 (48)		21 (34)	2.44 (0.64, 10.59)	
**Adjusted**^**b**^ **(N = 78)**						
**Vaginal microbiota**						
*Lactobacillus-*dominated		5 (26)		24 (41)	REF	
*L*. *iners*-dominated		6 (32)		14 (24)	1.35 (0.23, 7.95)	
Low-*Lactobacillus*		8 (42)		21 (36)	0.59 (0.09, 3.63)	
**Race**						
Other		3 (16)		30 (51)	REF	
Black or African American		16 (84)		29 (49)	5.07 (1.10, 33.27)	
**Hormonal Contraception**						
None		7 (37)		9 (15)	REF	
Non-hormonal		6 (32)		16 (27)	0.62 (0.10, 3.44)	
Hormonal		6 (32)		34 (58)	0.29 (0.06, 1.47)	
**Menstrual bleeding during sampling**						
No		8 (42)		32 (54)	REF	
Yes		11 (58)		27 (46)	2.43 (0.61, 10.84)	
**Longitudinal Vaginal Microbiota Profiles**	**Fibroid Number**			**3 or more Fibroids OR (95% CI)**	**1–2 Fibroids OR (95% CI)**	**No Fibroids OR (95% CI)**
	**3 or more Fibroids N (column %)**	**1–2 Fibroids N (column %)**	**No Fibroids N (column %)**			
**Unadjusted**^**c**^ **(N = 83)**						
*Lactobacillus-*dominated	2 (22)	3 (25)	26 (42)	REF	REF	REF
*L*. *iners*-dominated	2 (22)	4 (33)	15 (24)	1.71 (0.11, 25.90)	2.27 (0.33, 17.65)	REF
Low-*Lactobacillus*	5 (56)	5 (42)	21 (34)	3.03 (0.44, 34.90)	2.04 (0.35, 14.65)	REF
**Adjusted**^**d**^ **(N = 78)**						
*Lactobacillus-*dominated	2 (22)	3 (30)	24 (41)	REF	REF	REF
*L*. *iners*-dominated	2 (22)	4 (40)	14 (24)	0.51 (0.01, 14.34)	2.00 (0.27, 16.58)	REF
Low-*Lactobacillus*	5 (56)	3 (30)	21 (36)	0.26 (0.00, 5.79)	0.79 (0.07, 8.45)	REF
**Race**						
Other	0 (0)	3 (30)	30 (51)	REF	REF	REF
Black or African American	9 (100)	7 (70)	29 (49)	11.67 (2.03, infinity)	2.21 (0.40, 15.95)	REF
**Hormonal Contraception**						
None	5 (56)	2 (20)	9 (15)	REF	REF	REF
Non-hormonal	2 (22)	4 (40)	16 (27)	0.26 (0.01, 3.17)	1.10 (0.11, 15.61)	REF
Hormonal	2 (22)	4 (40)	34 (58)	0.16 (0.01, 1.34)	0.55 (0.06, 7.80)	REF
**Menstrual bleeding during sampling**						
No	2 (22)	6 (60)	32 (54)	REF	REF	REF
Yes	7 (78)	4 (40)	27 (46)	10.98 (1.07, 590.66)	1.08 (0.18, 6.28)	REF

Abbreviations: OR, odds ratio; CI, confidence interval; CST, community state type.

^a^Unadjusted odds ratio (OR) estimated using exact logistic regression

^b^Adjusted OR (aOR), estimated using exact logistic regression; adjusted for race, menstrual bleeding during sampling and type of contraception (hormonal, non-hormonal and none)

^c^OR, estimated using exact multinomial logistic regression

^d^aOR estimated using exact multinomial logistic regression; adjusted for race, menstrual bleeding during sampling and type of contraception (hormonal, non-hormonal and none)

## Discussion

In this preliminary study of 83 reproductive-age participants, *L*. *iners*-dominated and low-*Lactobacillus* longitudinal profiles of vaginal microbiota had higher odds of the presence of uterine fibroids in the unadjusted analyses, however findings were not statistically significant. Further, after adjustment for race, HC, and menstrual bleeding during sampling, there were no apparent associations. We investigated the *L*. *iners-*dominated longitudinal profile separately from the other *Lactobacillus*-dominated profiles because it is hypothesized that not all vaginal *Lactobacillus* spp. are equally protective; *L*. *iners* has been associated with increased BV and sexually transmitted infection incidence in comparison to *L*. *crispatus*-dominated vaginal microbiota [53, 77–80]. The reduced protective capabilities of *L*. *iners* may be due to its ability to only make L-lactic acid and its tendency to be a tipping point to BV states [25, 36, 81–83].

It has been hypothesized that reproductive tract infections, including CT and BV, may lead to a chronic pro-inflammatory state which could promote the development of uterine fibroids. However, initial epidemiologic studies of BV based on a single sample time point demonstrated conflicting results [32, 84, 85]. In a 2017 study of 660 African American women in the Study of Environment, Lifestyle, and Fibroids (SELF) cohort recruited in Detroit, Michigan, Moore *et al*. found self-reported history of BV at study baseline was associated with a 35% (aRR = 1.35, 95% CI: 0.93–1.95) increased risk for incident uterine fibroid detection and approximately 2-times increased risk for 3 or more uterine fibroids (aRR = 2.21, 95% CI 1.01–4.81) over the next 38 months (median) [32]. However, two recent sub-analyses of the SELF cohort study by Moore *et al*., in 2021 and 2022 respectively, found no association between baseline Nugent-BV [22] (BV assessed by Nugent’s Gram stain score [34]) (N = 197) or high relative abundance of eight BV-associated bacterial taxa (N = 1027) with incident uterine fibroid over a 5-year follow-up period [33, 35]. Epidemiologic studies of CT and uterine fibroids have shown mixed results as well. Faerstein *et al*. and Laughlin *et al*. reported non-significant positive associations between self-reported history of CT and uterine fibroid risk [86, 87]. A subsequent cross-sectional study (N = 1,587) of the SELF cohort by Moore *et al*. found a history of CT infection at baseline, determined with micro-immunofluorescence assays on serum, was inversely associated with uterine fibroid incidence (aOR = 0.80, 95% CI: 0.54–0.87) over a 5-year study period [88]. In contrast, a recent 2021 analysis by Moore and Baird found CT determined by seroprevalence was not associated with uterine fibroid development when using prospective TVUS data [89].

As this is an emerging topic, we sought to expand existing research by utilizing longitudinal profiles to characterize the microbiota and identify less optimal compositions, as in a *L*. *iners* and low-*Lactobacillus* dominated state, that may be associated with fibroid development. As it is well documented that the vaginal microbiota can fluctuate [36–39], a strength of this study was the use of longitudinal profiles to describe the vaginal microbiota. Another strength of this study was that we controlled for type of contraception *a priori* because past studies have shown associations between HC and fibroids as well as HC’s effect on the vaginal microenvironment, including altered bacterial composition and immune cell profiles [28, 90–92]. Results from research on associations of oral contraceptives and uterine fibroid development, including many large, longitudinal epidemiologic observational studies, report a decreased risk of uterine fibroids amongst oral contraceptive users [17, 30, 31, 71, 73, 93] Additionally, the use of depot medroxyprogesterone acetate (DMPA), a progestin contraceptive injection, has been shown in several studies to be protective against uterine fibroid development, with declining risk for longer duration of use [30, 94–96]. A study by Wise *et al*. found progestin-injectables were associated with a reduced risk of uterine fibroid incidence by 40% (95% CI: 0.4–0.9) [30]. It is theorized that the lower estradiol levels with the use of DMPA could prevent fibroid growth by down regulating the estrogen and progesterone receptors implicated in tumor cell proliferation [95, 97–99]. Although, observational studies indicate an association between progestin-only HC and reduced risk of uterine fibroids, more clinical trials are needed to determine whether DMPA should be considered an active therapy [100, 101].

There were limitations to this secondary analysis. The analysis was based on prevalent uterine fibroids at baseline (visit for TVUS), and therefore, we cannot determine factors associated with the initial development, nor the longitudinal growth of the uterine fibroids over time. This study was also limited in sample size, and much of the covariate information, such as type of contraception and severity of menstrual bleeding at baseline, was self-reported and missing in 10% and 39% of the study population, respectively. However, we were able to include HC in the analysis, an important variable that is linked to both BV and fibroid development. Limiting the control group to patients with no clinical findings on TVUS did not affect the results and suggest the control group was not biased by indication to TVUS. The Parent study did not collect information on endometriosis, which has been previously associated with symptomatic uterine fibroids [102]. Parity information was not collected as a part of this study, although pregnancy history, including never pregnant, at least one vaginal birth, at least one Cesarean section birth and at least one early pregnancy (loss/miscarriage/abortion) was assessed and determined not to confound our analyses.

There are also significant racial disparities that present obstacles to the study of fibroids. African American women have two-fold higher BV rates than white women [103, 104] and Black women have a higher incidence, number and volume of fibroids [105], with earlier onset, and more severe symptoms in comparison to other races [4, 106–108]. The etiology of racial disparities in uterine fibroids remains unknown, however mechanisms may include genetic predisposition, nutritional factors, environmental differences and chronic psychosocial stress [109–112]. In addition, Black women are also more likely to undergo invasive surgeries to treat uterine fibroids and have a higher risk for surgical complications than women of other races [113–116]. Large studies of uterine fibroid etiology have primarily utilized cohorts of Black women or have assessed race as an effect modifier for a more informative assessment [32, 33, 85, 88, 117–119]. We were unable to stratify by race in this study due to sample size, and instead controlled for it as a confounder in the final model. Other sources of chronic and acute inflammation in the reproductive tract may also contribute to uterine fibroids, such as intrauterine device (IUD), cesarean section, obesity, diet, aging, talc use, as well as environmental factors including reactive oxygen species and toxic metals [18, 84]. We were not able to assess these factors.

### Conclusion

This study did not find a statistically significant association between longitudinal profiles of vaginal microbiota and uterine fibroid case status after adjustment for common confounders (race, type of contraception, and menstrual bleeding during sampling). To our knowledge, this is the first study to use longitudinal profiles of the vaginal microbiota to assess associations with uterine fibroids, however it was limited by a small sample size. Future, larger studies which prospectively assess the development of uterine fibroids in the context of microenvironmental cofactors such as the vaginal microbiota, HC and host immune responses may be informative.

## Supporting information

S1 Fig Heatmap showing proportions of five community state types (CSTs) (I, II, III, IV, and V) within 83 participants over time which were clustered into longitudinal profiles. The color bar indicates longitudinal profiles designated LL, LI, LC, LG and LJ which were defined by clusters of proportions of the CSTs identified within a participant in the 1–2 weeks before TVUS. Longitudinal profile abbreviations: *LJ*, *Lactobacillus jensenii*; *LG*, *Lactobacillus gasseri*; *LI*, *Lactobacillus iners*; *LC*, *Lactobacillus crispatus*; *LL*, low-*Lactobacillus*.
(EPS)
