# Supplementary material for: Uterine fibroids and longitudinal profiles of the vaginal microbiota in a cohort presenting for transvaginal ultrasound
Source: PLoS One. 2024 Feb 5;19(2):e0296346. doi: 10.1371/journal.pone.0296346 (PMC10843103; doi:10.1371/journal.pone.0296346)
Supplement: Attachment — Submitted filename: Response_to_reviewers_5Dec2023.docx [file pone.0296346.s002.docx]

**Response to reviewers, PONE-D-23-15016**

**Uterine fibroids and longitudinal profiles of the vaginal microbiota in a cohort presenting for transvaginal ultrasound**

Thank you for the opportunity to revise our manuscript. Below are **reviewer comments (bold)** and our responses following. The line numbers correspond to the clean copy of the manuscript.

**3. We note that you have included the phrase “data not shown” in your manuscript. Unfortunately, this does not meet our data sharing requirements. PLOS does not permit references to inaccessible data. We require that authors provide all relevant data within the paper, Supporting Information files, or in an acceptable, public repository. Please add a citation to support this phrase or upload the data that corresponds with these findings to a stable repository (such as Figshare or Dryad) and provide and URLs, DOIs, or accession numbers that may be used to access these data. Or, if the data are not a core part of the research being presented in your study, we ask that you remove the phrase that refers to these data.**

Thank you for the opportunity to clarify. These data will be available at NCBI’s dbGAP (accession #: phs002211.v2.p1), but were not included in a table in the paper. We have clarified the text and the Results now read: “Black race, low income and menstrual bleeding during sampling were independently associated with a low-*Lactobacillus* longitudinal profile (all p<0.05).” (Line 237-239.)

**4. Please include your full ethics statement in the ‘Methods’ section of your manuscript file. In your statement, please include the full name of the IRB or ethics committee who approved or waived your study, as well as whether or not you obtained informed written or verbal consent. If consent was waived for your study, please include this information in your statement as well.**

We have clarified the text and it now reads: “This study was approved by the University of Maryland, Baltimore (UMB) Institutional Review Board, and informed written consent was obtained from each participant.” (Lines 143-144.)

**Reviewer #1:**

**-line 119: the authors state that women with STIs were not included in the study. Was Mycoplasma genitalium positivity considered or not? I have not seen this pathogen included in their list. If not, authors should comment why they have not performed Mgen laboratory tests.**

*Mycoplasma genitalium* (MG) screening is not part of routine clinical care in the U.S., and there is no evidence that MG impacts fibroids. The GALE study did not test for MG. Analyzing the 16S rRNA gene amplicon data for MG would not be informative as it is not a validated measure.

**- line 121: I am wondering why women with symptomatic BV condition have been excluded. I think that the authors should add comments in the methods.**

The GALE study excluded participants who had symptomatic BV at baseline because the study clinician would have prescribed antibiotics which can impact the vaginal microbiota in the week prior to TVUS. The Methods read: “Participants were excluded from the parent study if they were under age 18, diagnosed with pelvic inflammatory disease, cancers of the uterus, ovaries, or pelvic structures, prescribed antibiotics or antifungals within a month of starting the study, had an immune condition, were taking immunosuppressants, or self-reported lubricant use in the week prior to TVUS. (Lines 113-117).” We have also clarified the text in the Methods section, and it now reads: “Participants were also excluded if laboratory results confirmed *Neisseria gonorrhea*, CT or *Trichomonas vaginalis* (BD MAX CT/GC/TV), HIV (Abbot ARCHITECT HIV Ag/Ab Combo), syphilis (BD Macro-Vue), pregnancy or were diagnosed with symptomatic BV or vulvovaginal candidiasis (VVC) at the baseline visit of the parent study. Those diagnosed with symptomatic BV at the baseline clinic visit were prescribed antibiotics and were therefore excluded from the parent study.” (Lines 118-123.)

**- line 182 and table 1: for most European readers the difference between ethnicity and race can be confusing. Please expand this part.**

Among the 83 participants, 59% were Black/African American. Due to limited sample size, analyses were dichotomized to Black/African American or white and Asian for all modeling. (Lines 188-191.) For clarity, we are now only reporting self-reported race (Asian, Native American or Alaskan Native, Native Hawaiian or Pacific Islander, Black or African American, white or other).

**- line 231: Black race and low income were significantly associated with low Lactobacillus profiles. I am wondering if Black race and low income are significantly associated or not. If these are associated the authors should revise the sentence.**

Black race and low income were independently associated with the vaginal microbiota, but income was not associated with fibroids (Lines 237-239). Race and income were associated with each other in this population (p<0.01). We are not completely clear what the reviewer is asking, but we have added p-values in the Results section to help add clarity. We have revised the sentence in the Results “Black race, low income and menstrual bleeding during sampling were all independently associated with a low-*Lactobacillus* longitudinal profile (all p<0.05).” (Lines 237-239.)

**- The discussion is quite long and could be shortened**

We have reviewed the discussion and feel that it presents a concise presentation of the conflicting data available and provides information for future work. If the editor would like us to trim further, we can certainly revise again.

**Reviewer #2: Review:
This is study of the vaginal microbiome in patients with benign gynaecological conditions. Although it is limited in sample size, the hypothesis and urgency is clear. Especially the remarkable frequency of uterine fibroids in ethnically african women compared to white women raises the question whether the higher prevalence of BV in this group is associated with uterine fibroids.**

**One of the weaknesses of this study is the heterogeneity of the control group. These women may also have a condition that causes pelvic mass or pain in which vaginal microbiome also plays a role. Is there a way to put these data in the context of other longitudinal datasets of vaginal microbiome in patients or control group from past studies. How do these compare to the study group here, cases and controls taken together? Do women who get a referral for TVUS due to pelvic symptoms have a higher incidence of low-Lactobacillus microbiota? This could place the relatively small group of women with uterine fibroids in context.**

The reviewer is correct that the findings may be biased because controls also had a referral indication for TVUS, however, a referral for “pelvic symptoms” was not associated with vaginal microbiota outcomes (p=0.8). Further, “evaluation of abnormal uterine bleeding” (p=0.7) and “assessment of a pelvic mass” (p=0.1) were also not significantly associated with low-*Lactobacillus* vaginal microbiota. In addition, if we limit controls to only those 32 patients who had no clinical findings on TVUS, the findings remain similar.

**Line 123
This line is not clear. Do I understand correctly that the exclusion criteria for the cases group were different than for the control group to increase the number of cases? If so, please clarify which exclusion/inclusion criteria were used for the cases and control groups? This may explain some of the observed demographic/ethnic differences.**

This secondary analysis included all individuals who presented for TVUS, had sonography reports and were reproductive age. Cases were defined as those whose TVUS report indicated uterine fibroids and controls were those whose report did not indicate uterine fibroids (but may have other findings including cysts and adenomyosis). There were not differential exclusion criteria for cases and controls. Exclusion criteria are listed above and on lines (113-123) in the manuscript. The Methods now reads “Controls were included even if they had other findings (cysts and adenomyosis).” (Lines 126-127).

**Reviewer #3: Title: Uterine fibroids and longitudinal profiles of the vaginal microbiota in a cohort presenting for transvaginal ultrasound

Here a secondary analysis was performed on vaginal microbiota data collected from women participating in the GALE study with the aim to assess whether the vaginal microbiota composition associated with having uterine fibroids while controlling for important confounding factors such as oral contraceptive use. The authors use sound, previously well described, analytical methods to reduce the microbiota data into biologically and clinically relevant bacterial groups. They further collapse longitudinal microbiome datasets to allow for binomial statistical modelling. The authors do not detect any statistically significant relationship between having uterine fibroids and the vaginal microbiota composition. Their study has a number of limitations all of which are addressed in the manuscript and mainly arise from this being a secondary analysis and only few women in the parent study had uterine fibroids. Nonetheless, this explorative study provides useful insights for future work.**

Thank you very much for your feedback.
