# Supplementary material for: Uterine fibroids and longitudinal profiles of the vaginal microbiota in a cohort presenting for transvaginal ultrasound
Source: PLoS One. 2024 Feb 5;19(2):e0296346. doi: 10.1371/journal.pone.0296346 (PMC10843103; doi:10.1371/journal.pone.0296346)
Supplement: Peer review history

# Peer review: Decision Letter 0

**DOI:** 10.1371/journal.pone.0296346.r001

**Authors:** Sylvia Maria Bruisten

13 Jun 2023

PONE-D-23-15016Uterine fibroids and longitudinal profiles of the vaginal microbiota in a cohort presenting for transvaginal ultrasoundPLOS ONE

Dear Dr. Brotman,

Thank you for submitting your manuscript to PLOS ONE. After careful consideration, we feel that it has merit but does not fully meet PLOS ONE’s publication criteria as it currently stands. Therefore, we invite you to submit a revised version of the manuscript that addresses the points raised during the review process.

Please submit your revised manuscript by Jul 28 2023 11:59PM. If you will need more time than this to complete your revisions, please reply to this message or contact the journal office at plosone@plos.org. When you're ready to submit your revision, log on to https://www.editorialmanager.com/pone/ and select the 'Submissions Needing Revision' folder to locate your manuscript file.

Please include the following items when submitting your revised manuscript:

- A rebuttal letter that responds to each point raised by the academic editor and reviewer(s). You should upload this letter as a separate file labeled 'Response to Reviewers'.
- A marked-up copy of your manuscript that highlights changes made to the original version. You should upload this as a separate file labeled 'Revised Manuscript with Track Changes'.
- An unmarked version of your revised paper without tracked changes. You should upload this as a separate file labeled 'Manuscript'.

If you would like to make changes to your financial disclosure, please include your updated statement in your cover letter. Guidelines for resubmitting your figure files are available below the reviewer comments at the end of this letter.

If applicable, we recommend that you deposit your laboratory protocols in protocols.io to enhance the reproducibility of your results. Protocols.io assigns your protocol its own identifier (DOI) so that it can be cited independently in the future. For instructions see: https://journals.plos.org/plosone/s/submission-guidelines#loc-laboratory-protocols. Additionally, PLOS ONE offers an option for publishing peer-reviewed Lab Protocol articles, which describe protocols hosted on protocols.io. Read more information on sharing protocols at https://plos.org/protocols?utm_medium=editorial-email&utm_source=authorletters&utm_campaign=protocols.

We look forward to receiving your revised manuscript.

Kind regards,

Sylvia Maria Bruisten, Ph.D

Academic Editor

PLOS ONE

Journal Requirements:

When submitting your revision, we need you to address these additional requirements.

1. Please ensure that your manuscript meets PLOS ONE's style requirements, including those for file naming. The PLOS ONE style templates can be found at 

https://journals.plos.org/plosone/s/file?id=wjVg/PLOSOne_formatting_sample_main_body.pdf and 

https://journals.plos.org/plosone/s/file?id=ba62/PLOSOne_formatting_sample_title_authors_affiliations.pdf

2. Thank you for stating the following in the Competing Interests section: 

   "I have read the journal's policy and the authors of this manuscript have the following competing interests: ST has been a consultant for Biofire Diagnostics, Roche Molecular Diagnostics and Luca Biologics, receives royalties from UPTODATE and has received speaker honoraria from Roche Molecular Diagnostics and Medscape."

Please confirm that this does not alter your adherence to all PLOS ONE policies on sharing data and materials, by including the following statement: "This does not alter our adherence to  PLOS ONE policies on sharing data and materials.” (as detailed online in our guide for authors http://journals.plos.org/plosone/s/competing-interests).  If there are restrictions on sharing of data and/or materials, please state these. Please note that we cannot proceed with consideration of your article until this information has been declared. 

Please include your updated Competing Interests statement in your cover letter; we will change the online submission form on your behalf.

3. We note that you have included the phrase “data not shown” in your manuscript. Unfortunately, this does not meet our data sharing requirements. PLOS does not permit references to inaccessible data. We require that authors provide all relevant data within the paper, Supporting Information files, or in an acceptable, public repository. Please add a citation to support this phrase or upload the data that corresponds with these findings to a stable repository (such as Figshare or Dryad) and provide and URLs, DOIs, or accession numbers that may be used to access these data. Or, if the data are not a core part of the research being presented in your study, we ask that you remove the phrase that refers to these data.

4. Please include your full ethics statement in the ‘Methods’ section of your manuscript file. In your statement, please include the full name of the IRB or ethics committee who approved or waived your study, as well as whether or not you obtained informed written or verbal consent. If consent was waived for your study, please include this information in your statement as well. 

5. Please review your reference list to ensure that it is complete and correct. If you have cited papers that have been retracted, please include the rationale for doing so in the manuscript text, or remove these references and replace them with relevant current references. Any changes to the reference list should be mentioned in the rebuttal letter that accompanies your revised manuscript. If you need to cite a retracted article, indicate the article’s retracted status in the References list and also include a citation and full reference for the retraction notice.

Additional Editor Comments:

The three reviewers are quite positive over this manuscript with which I agree. I have no further comments.

[Note: HTML markup is below. Please do not edit.]

Reviewers' comments:

Reviewer's Responses to Questions

**Comments to the Author**

1. Is the manuscript technically sound, and do the data support the conclusions?

The manuscript must describe a technically sound piece of scientific research with data that supports the conclusions. Experiments must have been conducted rigorously, with appropriate controls, replication, and sample sizes. The conclusions must be drawn appropriately based on the data presented. 

Reviewer #1: Yes

Reviewer #2: Yes

Reviewer #3: Yes

**********

2. Has the statistical analysis been performed appropriately and rigorously? 

Reviewer #1: Yes

Reviewer #2: Yes

Reviewer #3: Yes

**********

3. Have the authors made all data underlying the findings in their manuscript fully available?

The PLOS Data policy requires authors to make all data underlying the findings described in their manuscript fully available without restriction, with rare exception (please refer to the Data Availability Statement in the manuscript PDF file). The data should be provided as part of the manuscript or its supporting information, or deposited to a public repository. For example, in addition to summary statistics, the data points behind means, medians and variance measures should be available. If there are restrictions on publicly sharing data—e.g. participant privacy or use of data from a third party—those must be specified.

Reviewer #1: Yes

Reviewer #2: Yes

Reviewer #3: Yes

**********

4. Is the manuscript presented in an intelligible fashion and written in standard English?

PLOS ONE does not copyedit accepted manuscripts, so the language in submitted articles must be clear, correct, and unambiguous. Any typographical or grammatical errors should be corrected at revision, so please note any specific errors here.

Reviewer #1: Yes

Reviewer #2: Yes

Reviewer #3: Yes

**********

5. Review Comments to the Author

Please use the space provided to explain your answers to the questions above. You may also include additional comments for the author, including concerns about dual publication, research ethics, or publication ethics. (Please upload your review as an attachment if it exceeds 20,000 characters)

Reviewer #1: The interesting manuscript by Robbins et al. tries to shed light on the association between uterine fibroids and vaginal bacterial composition. Anyway, it seems that no particular association can be found between vaginal microbiome profiles and uterine fibroids cases.

I have just few questions for the authors:

-line119: the authors state that women with STIs were not included in the study. Was Mycoplasma genitalum positivity considered or not? I have not seen this pathogen included in their list. If not, authors should comment why they have not performed Mgen laboratory tests.

- line 121: I am wondering why women with symptomatic BV condition have been excluded. I Think that the authors should add comments in the methods.

- line 182 and table 1: for most European readers the difference between ethnicity and race can be confusing. Please expand this part.

- line 231: Black race and low income were significantly associated with low Lactobacillus profiles. I am wondering if Black race and low income are significantly associated or not. If these are associated the authors should revise the sentence.

- The discussion is quite long and could be shortened

Reviewer #2: Review:

This is study of the vaginal microbiome in patients with benign gynaecological conditions. Although it is limited in sample size, the hypothesis and urgency is clear. Especially the remarkable frequency of uterine fibroids in ethnically african women compared to white women raises the question whether the higher prevalence of BV in this group is associated with uterine fibroids.

One of the weaknesses of this study is the heterogeneity of the control group. These women may also have a condition that causes pelvic mass or pain in which vaginal microbiome also plays a role. Is there a way to put these data in the context of other longitudinal datasets of vaginal microbiome in patients or control group from past studies. How do these compare to the study group here, cases and controls taken together? Do women who get a referral for TVUS due to pelvic symptoms have a higher incidence of low-Lactobacillus microbiota? This could place the relatively small group of women with uterine fibroids in context.

Line 123

This line is not clear. Do I understand correctly that the exclusion criteria for the cases group were different than for the control group to increase the number of cases? If so, please clarify which exclusion/inclusion criteria were used for the cases and control groups? This may explain some of the observed demographic/ethnic differences.

Reviewer #3: Title: Uterine fibroids and longitudinal profiles of the vaginal microbiota in a cohort presenting for transvaginal ultrasound

Here a secondary analysis was performed on vaginal microbiota data collected from women participating in the GALE study with the aim to assess whether the vaginal microbiota composition associated with having uterine fibriods while controlling for important confounding factors such as oral contraceptive use. The authors use sound, previously well described, analytical methods to reduce the microbiota data into biologically and clinically relevant bacterial groups. They further collapse longitudinal microbiome datasets to allow for binomial statistical modelling. The authors do not detect any statistically significant relationship between having uterine fibroids and the vaginal microbiota composition. Their study has a number of limitations all of which are addressed in the manuscript and mainly arise from this being a secondary analaysis and only few women in the parent study had uterine fibroids. Nonetheless, this explorative study provides useful insights for future work.

**********

6. PLOS authors have the option to publish the peer review history of their article (what does this mean?). If published, this will include your full peer review and any attached files.

If you choose “no”, your identity will remain anonymous but your review may still be made public.

**Do you want your identity to be public for this peer review?** For information about this choice, including consent withdrawal, please see our Privacy Policy.

Reviewer #1: **Yes: **Antonella Marangoni

Reviewer #2: No

Reviewer #3: No

**********

[NOTE: If reviewer comments were submitted as an attachment file, they will be attached to this email and accessible via the submission site. Please log into your account, locate the manuscript record, and check for the action link "View Attachments". If this link does not appear, there are no attachment files.]

While revising your submission, please upload your figure files to the Preflight Analysis and Conversion Engine (PACE) digital diagnostic tool, https://pacev2.apexcovantage.com/. PACE helps ensure that figures meet PLOS requirements. To use PACE, you must first register as a user. Registration is free. Then, login and navigate to the UPLOAD tab, where you will find detailed instructions on how to use the tool. If you encounter any issues or have any questions when using PACE, please email PLOS at figures@plos.org. Please note that Supporting Information files do not need this step.

# Author comment: Author response to Decision Letter 0

**DOI:** 10.1371/journal.pone.0296346.r002

5 Dec 2023

See response to reviewers in the "attach files" section.

Attachment Submitted filename: Response_to_reviewers_5Dec2023.docx Click here for additional data file.

# Editor report: Decision Letter 1

**DOI:** 10.1371/journal.pone.0296346.r003

**Authors:** Sylvia Maria Bruisten

11 Dec 2023

Uterine fibroids and longitudinal profiles of the vaginal microbiota in a cohort presenting for transvaginal ultrasound

PONE-D-23-15016R1

Dear Dr. Brotman,

We’re pleased to inform you that your manuscript has been judged scientifically suitable for publication and will be formally accepted for publication once it meets all outstanding technical requirements.

Within one week, you’ll receive an e-mail detailing the required amendments. When these have been addressed, you’ll receive a formal acceptance letter and your manuscript will be scheduled for publication.

An invoice for payment will follow shortly after the formal acceptance. To ensure an efficient process, please log into Editorial Manager at http://www.editorialmanager.com/pone/, click the 'Update My Information' link at the top of the page, and double check that your user information is up-to-date. If you have any billing related questions, please contact our Author Billing department directly at authorbilling@plos.org.

If your institution or institutions have a press office, please notify them about your upcoming paper to help maximize its impact. If they’ll be preparing press materials, please inform our press team as soon as possible -- no later than 48 hours after receiving the formal acceptance. Your manuscript will remain under strict press embargo until 2 pm Eastern Time on the date of publication. For more information, please contact onepress@plos.org.

Kind regards,

Sylvia Maria Bruisten, Ph.D

Academic Editor

PLOS ONE

Additional Editor Comments (optional):

All comments were answered well. There is only one remark that was also raised by reviewer #1.

In the United States 'race' is used whereas this has a negative connotation in Europe (and maybe elsewhere).

For future studies the authors should be aware that 'ethinicity' is the preferred term instead of 'race'.

Reviewers' comments:

# Editor report: Acceptance letter

**DOI:** 10.1371/journal.pone.0296346.r004

**Authors:** Sylvia Maria Bruisten

26 Jan 2024

PONE-D-23-15016R1 

PLOS ONE

Dear Dr. Brotman, 

I'm pleased to inform you that your manuscript has been deemed suitable for publication in PLOS ONE. Congratulations! Your manuscript is now being handed over to our production team.

At this stage, our production department will prepare your paper for publication. This includes ensuring the following:

* All references, tables, and figures are properly cited

* All relevant supporting information is included in the manuscript submission,

* There are no issues that prevent the paper from being properly typeset

If revisions are needed, the production department will contact you directly to resolve them. If no revisions are needed, you will receive an email when the publication date has been set. At this time, we do not offer pre-publication proofs to authors during production of the accepted work. Please keep in mind that we are working through a large volume of accepted articles, so please give us a few weeks to review your paper and let you know the next and final steps. 

Lastly, if your institution or institutions have a press office, please let them know about your upcoming paper now to help maximize its impact. If they'll be preparing press materials, please inform our press team within the next 48 hours. Your manuscript will remain under strict press embargo until 2 pm Eastern Time on the date of publication. For more information, please contact onepress@plos.org.

If we can help with anything else, please email us at customercare@plos.org.

Thank you for submitting your work to PLOS ONE and supporting open access. 

Kind regards, 

PLOS ONE Editorial Office Staff

on behalf of

Dr. Sylvia Maria Bruisten 

Academic Editor

PLOS ONE
